# Phenotype and Hierarchy of Two Transgenic T Cell Lines Targeting the Respiratory Syncytial Virus K^d^M2_82-90_ Epitope Is Transfer Dose-Dependent

**DOI:** 10.1371/journal.pone.0146781

**Published:** 2016-01-11

**Authors:** Kaitlyn M. Morabito, Noam Erez, Barney S. Graham, Tracy J. Ruckwardt

**Affiliations:** 1 Vaccine Research Center, National Institute of Allergy and Infectious Disease, National Institutes of Health, Bethesda, Maryland, United States of America; 2 Department of Microbiology and Immunology, Georgetown University Medical Center, Washington, DC, United States of America; 3 Department of Infectious Diseases, Israel Institute for Biological Research, Ness-Ziona, Israel; University of Iowa, UNITED STATES

## Abstract

In this study, we compared two lines of transgenic CD8+ T cells specific for the same K^d^M2_82-90_ epitope of respiratory syncytial virus in the CB6F1 hybrid mouse model. Here we found that these two transgenic lines had similar *in vivo* abilities to control viral load after respiratory syncytial virus infection using adoptive transfer. Transfer of the TRBV13-2 line resulted in higher levels of IL-6 and MIP1-α in the lung than TRBV13-1 transfer. Interestingly, when large numbers of cells were co-transferred, the lines formed a hierarchy, with TRBV13-2 being immunodominant over TRBV13-1 in the mediastinal lymph node despite no identifiable difference in proliferation or apoptosis between the lines. This hierarchy was not established when lower cell numbers were transferred. The phenotype and frequency of proliferating cells were also cell transfer dose-dependent with higher percentages of CD127^lo^CD62L^lo^KLRG1^lo^ and proliferating cells present when lower numbers of cells were transferred. These results illustrate the importance of cell number in adoptive transfer experiments and its influence on the phenotype and hierarchy of the subsequent T cell response.

## Introduction

CD8+ cytotoxic T lymphocytes (CTLs) are crucial for an efficient immune response to viral infection and play a major role in viral clearance. Infection leads to presentation of peptides from the pathogen on the major histocompatibility complex (MHC) class I molecules of infected cells (pMHC) which interacts with the T cell receptor (TCR) of virus-specific CTL. TCR recognition of the pMHC leads to T cell activation driving cytokine secretion, cytolytic activity, and proliferation. Variable recognition and expansion of CTLs targeting different viral epitopes leads to the generation of an immunodominance hierarchy [1]. In addition to immunodominance hierarchy, the finding that CTLs with multiple TCRs react with the same T cell epitope illustrates the complexity of the CTL immune response [2]. While several mechanisms contributing to the immunodominance hierarchy of T cell epitopes have been elucidated [3], the coordinate regulation of different T cell lines responding to the same epitope in the context of a viral infection has not yet been thoroughly studied.

Past studies have demonstrated the importance of CTLs in respiratory syncytial virus (RSV) clearance [4] as well as their role and involvement in immunopathology [5, 6]. In the hybrid CB6F1 mouse, RSV infection elicits responses to two major CD8+ T cell epitopes: K^d^M2_82–90_ and D^b^M_187–195_ [7]. While the immune response to K^d^M2_82–90_ is manifested by rapid proliferation and cytokine secretion, the immune response to the D^b^M_187–195_ epitope is characterized by higher functional avidity, superior cytolytic activity and enhanced viral clearance [8]. In a recent study, we described the generation of two transgenic T cell (TCR Tg) lines specific for the dominant K^d^M2_82–90_ epitope. These TCR Tg lines were selected as representatives of abundant (“public”) and rare (“private”) T cells and designated by their V beta gene usage as TRBV13-1 and TRBV13-2, respectively. The prior study characterized and compared the functional profile of these two novel TCR transgenic strains and was the first example of two different TCR Tg lines specific for same epitope with distinct features. We showed that cells from both TCR Tg lines exhibited similar functional avidity according to their proliferation and cytokine secretion properties *in vitro* in response to peptide stimulation. Some minor differences between the two lines on a BALB/c background were observed, suggesting that the nature of a specific CTL response can vary from one line to another [9]. While the BALB/c strain allows the study of H-2^d^-restricted CD8+ T cell responses, the CB6F1 hybrid strain allows the study of H-2^b^ and H-2^d^- restricted CD8+ T cell responses simultaneously. We have extensively studied RSV infection in CB6F1 mice and are expanding the toolbox of reagents that can be used to study antigen presentation and immune responses in this strain.

In the present study, we extend the prior *in vitro* studies and evaluated *in vivo* characteristics of these lines in the CB6F1 murine model and specifically investigated relative properties of these cells *in vivo* when co-transferred into mice. Here, we show that adoptive transfer of TRBV13-1 resulted in lower levels of IL-6 and MIP-1α in the lungs of RSV-infected mice in comparison to TRBV13-2 transfer. However, transfer of TRBV13-1 alone or co-transfer of TRBV13-1 and TRBV13-2 induced more morbidity in infected animals compared to transfer of TRBV13-2 cells alone. Interestingly, although we did not find any differences in the proliferation or surface phenotype of the two Tg lines following transfer to CB6F1 mice, when high numbers of these Tg lines were co-transferred, TRBV13-2 cells numerically dominated TRBV13-1 cells in the mediastinal lymph node (MLN). This skewing was not observed when low numbers of cells were transferred. The effector phenotype of transferred cells also depended on the dose of transferred cells, with a transfer of lower numbers driving more cells toward an effector phenotype at the site of infection. These results further demonstrate the diversity and complexity of the T cell response in regards to the abundance and function of T cells with different specificities, but also of different T cell lines specific for the same pMHC complex.

## Materials and Methods

### Ethics statement

All mice used in this study and analysis were maintained according to the guidelines of the NIH Guide to the Care and Use of Laboratory Animals and the approval of the Animal Care and Use Committee of the Vaccine Research Center (VRC), National Institute of Allergy and Infectious Diseases at the National Institutes of Health. All mice were housed in a facility fully accredited by the Association for Assessment and Accreditation of Laboratory Animal Care International (AAALAC). All procedures were approved under animal care and use protocol numbers VRC-11-345 and VRC-14-466 and were conducted in strict accordance with all relevant federal and National Institutes of Health guidelines and regulations.

### Mice

CB6F1 mice were bred in house by crossing BALB/c mice with C57BL/6 or purchased from Jackson Labs (Bar Harbor, ME). Generation of TCR Tg TRBV13-1 and TRBV13-2 was described previously [9]. The TCR-Thy1.1+ transgenic mice (designated TRBV13-1 and TRBV13-2) were produced by crossing male parental TRBV13-1 and TRBV13-2 on a BALB/c background with female Thy1.1^+^ C57BL/6 mice (Jackson Labs, Bar Harbor, ME).

### Adoptive transfers of TCR Tg CD8+ T cells and RSV infection

CD8+ T cell transgenic T cells were isolated from the spleens of TRBV13-1 or TRBV13-2 mice using a CD8+ T cell isolation kit (Miltenyi) for untouched isolation prior to intravenous (IV) transfer into recipient CB6F1.

The indicated number of cells were transferred IV into each recipient one day prior to intranasal (IN) infection with 2x10^6^ PFU of RSV A2 generated as previously described [10]. At indicated time points post-infection mice were sacrificed and MLN and lungs were removed for further processing described below.

For BrdU incorporation studies, mice were intraperitoneal (IP) injected with 2mg of BrdU. Two hours later, animals were sacrificed and their lungs and MLN were removed and processed.

To evaluate *in vivo* proliferation, isolated CD8+ transgenic T cells were labeled with 5μM CellTrace Violet (Life technologies) and transferred into recipient mice as described above. The percent of the original population that divided in the MLN was calculated using the proliferation analysis module within the FlowJo software.

### Flow Cytometry

All washing procedures were done using flow buffer composed of PBS, 2%FBS and 0.05% NaN_3_. The following mAb clones were used for staining: CD3 (145-2C11), CD8 (2.43), Thy1.1 (HIS51), CXCR3 (CXCR3-173), CD62L (MEL-14), Vβ8.1/8.2 (MR5-2), Vβ8.3 (1B3.3), CD127 (A7R34), CD44 (IM7), and KLRG1 (2F1/KLRG1). All antibodies were purchased from eBioscience (San Diego, CA), BD Biosciences (San Diego, CA) or Biolegend (San Diego, CA). For tetramer analysis, cells were surface stained with K^d^M2_82-90_ conjugated to APC (Beckman Coulter, San Diego, CA) or K^d^M2_82-90_ conjugated to BV421. BrdU incorporation was determined using the BrdU staining kit from eBioscience according to the manufacturer protocol.

Spleen, lung and MLN tissues were harvested at the indicated times post-infection. Spleen and lung tissues were processed using a GentleMACS machine (Miltenyi). Mononuclear cells were purified using Fico-LITE at room temperature, washed, then resuspended in flow buffer. MLN tissues were ground between the ends of two frosted glass slides and mononuclear cells were isolated as described above. Staining for extracellular markers was done using the antibodies listed above for 20 minutes at 4°C. Samples were collected on an LSR-II flow cytometer (BD, San Jose, CA) and data were analyzed using FlowJo (Tristar, CA). Transferred CD8+ T cells were identified by expression of CD3, CD8, and Thy1.1.

### *in vivo* CTL assay

Target CB6F1 splenocytes were harvested from naïve CB6F1, purified as described above and labelled with either 5μM (high) or 0.25μM (low) CFSE followed by 3 washes with lymphocyte medium (RPMI, 10%FBS, 2 mM Glutamine, 1 mM Sodium Pyruvate, non-essential amino acids, 25 mM HEPES, 5×10^−5^ M β-mercaptoethanol and Pen/Strep antibiotics). CFSE^high^ labeled cells were loaded with K^d^M2_82-90_ at desired concentration and CFSE^low^ labeled cells were loaded with 10^-6^M K^d^–binding influenza virus A/Puerto Rico/8/34 NP_147–155_ (TYQRTRALV) peptide as an internal control. CFSE^high^ and CFSE^low^ target cells were washed 3 times and mixed at 1:1 ratio.

For *in vivo* CTL assays, 10^6^ target cells were transferred by IN instillation. Four hours later animals were sacrificed and their lungs were processed as described above for cell isolation. CFSE^high^ and CFSE^low^ labeled cells were counted by flow cytometry and specific cell lysis was calculated using the following formula:

$$\%\;specific\;lysis=\left(1-\frac{ratioX}{ratio\;control}\right)*100$$

where *ratioX* is $\frac{CFSEhigh}{CFSElow}$ in each sample and *ratio control* is $\frac{CFSEhigh}{CFSElow}$ of control uninfected mice.

### Plaque assay

Viral loads in the lungs of infected mice were determined as described previously [11]. Briefly, organs were quick frozen in MEM supplemented with 10% FBS, 2 mM Glutamine, and Pen/Strep antibiotics and stored at -80°C. Organs were quick-thawed and disassociated using a GentleMACs machine. Suspensions were centrifuged and supernatant used for the plaque assay on HEp-2 cells. Following a four day incubation, plates were fixed and H&E stained prior to counting plaques.

### Cytokine and chemokine analysis

Lungs from infected mice were removed and treated as described for plaque assay. Clarified supernatants were shipped to AssayGate (Ijamsville, MD) to determine protein concentrations by multiplex cytokine analysis.

### Statistical analysis

Statistical analysis was performed using t-test, one-way or two-way analysis of variance (ANOVA) and recommended post-tests in GraphPad Prism version 6 (GraphPad Software, San Diego, CA). Statistical significance is indicated by stars where **** stands for P<0.0001, *** for P<0.001, ** for P<0.01 and * for P<0.05.

## Results

### TRBV13-1 and TRBV13-2 exhibit similar efficiency in cytolytic activity *in vivo*

**Fig 1:**
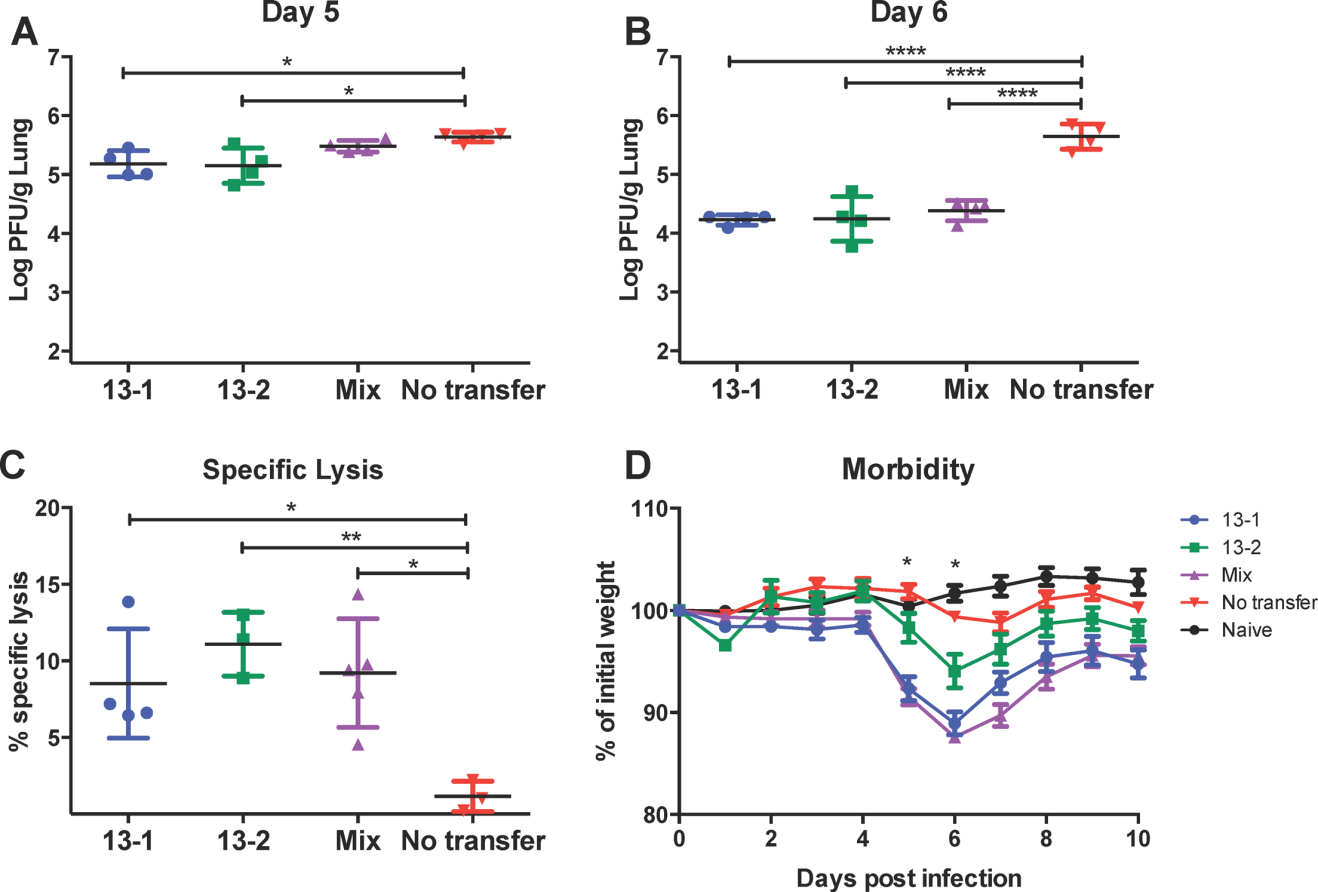
Transfer of TRBV13-1, TRBV13-2, and 1:1 mix leads to increased specific lysis, decreased viral loads, and increased morbidity after RSV infection. (A-D) CB6F1 mice received 10^6^ of either TRBV13-1 or TRBV13-2 cells or a 1:1 mixture of both (mix) one day prior to infection with RSV. On day 5 (A) and 6 (B) post-infection lungs were removed and processed to determine viral titer in the lungs by plaque assay. (C) CFSE labeled target cells were transferred by IN instillation into TRBV13-1 and TRBV13-2 recipient mice to evaluate cytolysis in the lung on day 4 post-infection. (D) Morbidity of mice in the different groups was evaluated by calculation of weight loss. * indicates days in which TRBV13-1 and Mix were significantly different than TRBV13-2.

In order to evaluate the cytolytic efficiency of these two Tg T cells, we transferred 10^6^ TRBV13-1 cells or TRBV13-2 or a 1:1 mixture (mix) of both into recipient CB6F1 by IV injection one day prior to infection with RSV. At three time points post-infection, lungs were harvested from infected mice and viral load was determined by plaque assay. No difference in viral clearance was apparent on day 4 post-infection in comparison to infected mice that did not receive adoptive transfer (data not shown). However, beginning at day 5 post-infection (Fig 1A) a significant reduction in viral load was seen in the lungs of mice that received either TRBV13-1 or TRBV13-2 in comparison to control infected animals (p<0.05). This trend continued on day 6 where all groups that received transgenic T cells by adoptive transfer exhibited a substantial and significant 1.5 Log reduction in viral load (Fig 1B, p<0.0001).

To measure the direct cytolytic activity, we transferred naïve Tg T cells into mice one day prior to infection with RSV. On day 4 post-infection, we performed an *in vivo* CTL assay by transferring CFSE-labeled target cells that were loaded with M2 peptide or control cells as described in the Materials and Methods. Four hours later, we harvested the lungs, processed them and determined the specific cytolytic activity of the adoptively transferred cells in each group. There was no significant difference between the number of TRBV13-1 and TRBV13-2 in the lungs (data not shown). All groups that received either TRBV13-1 or TRBV13-2 or both exhibited a significant higher cytolysis levels than the endogenous cytolysis response of the control group (p<0.05), but there was no difference between groups that received TRBV13-2 and TRBV13-1 (Fig 1C).

### Adoptive transfer of TRBV13-1 cells induces more severe morbidity in RSV infected mice

Activated CD8+ T cells are crucial for viral clearance from infected tissues. However, recruitment of highly activated T cells to the lungs can also cause morbidity due to the activity of effector cells [8, 12, 13]. In order to study the effect of the two different M2-specific lines on illness following RSV infection, we transferred TRBV13-1 cells or TRBV13-2 cells or a 1:1 mixture into mice, infected them with RSV and followed their weight loss as a marker for morbidity (Fig 1D). In the two weeks following infection, control mice that were infected with RSV but received no T cell transfer exhibited limited weight loss of not more than 2% of their initial weight (4% of maximal weight). On day 5 and 6 post-infection, animals that received TRBV13-1 alone or in combination with TRBV13-2 exhibited significantly more weight loss than mice that received TRBV13-2 alone (p<0.0001). Groups that received TRBV13-1 were also slower to recover and their weight remained low in comparison to TRBV13-2, which returned to their initial weight by day 9 post-infection. These data indicate that TRBV13-1 Tg cells cause more weight loss after RSV infection than TRBV13-2 Tg cells.

### TRBV13-2 Tg cells secrete higher levels of IL-6 and MIP-1α

**Fig 2:**
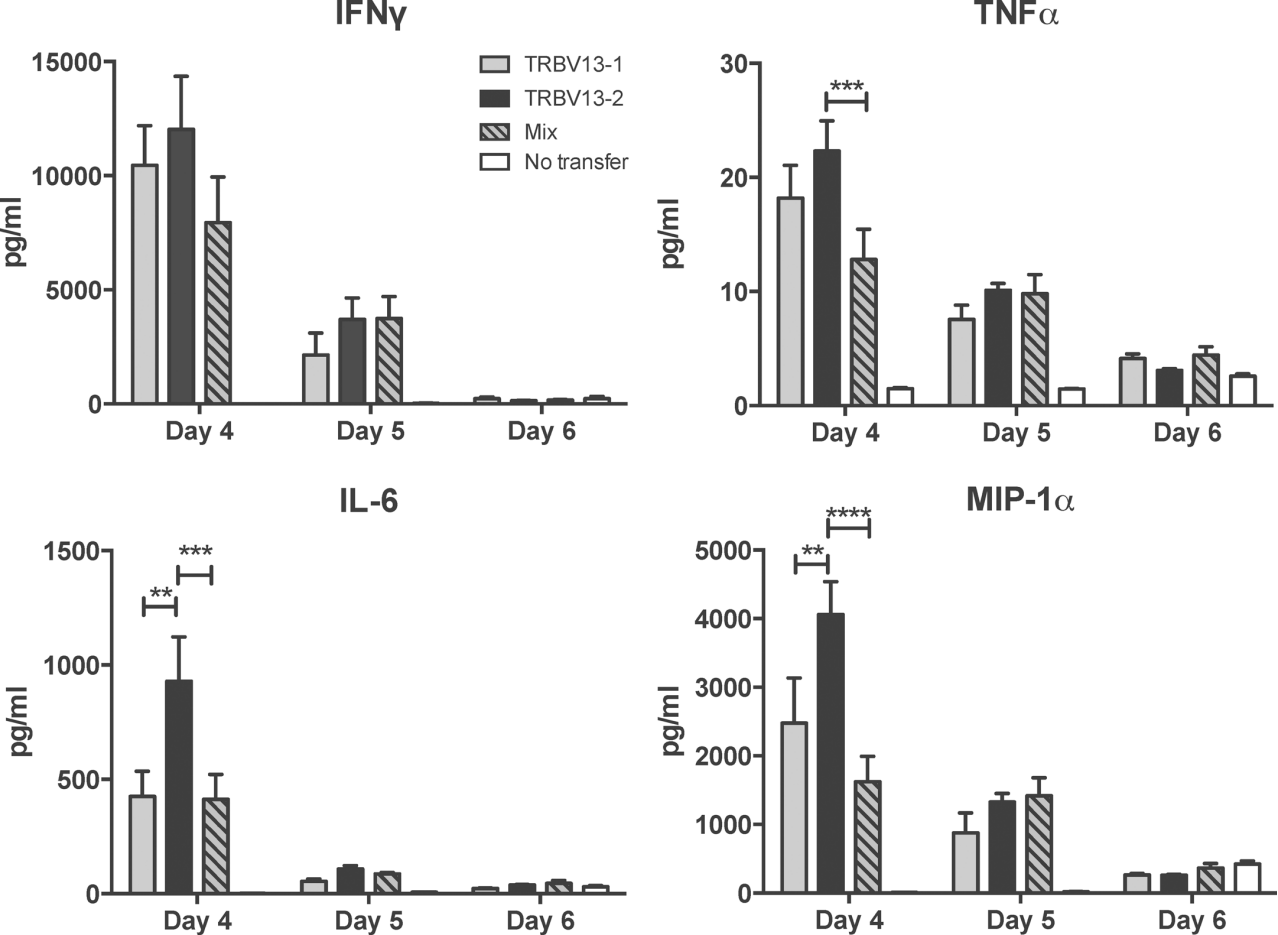
Transfer of TRBV 13–2 leads to increased levels of proinflammatory cytokines in the lungs following RSV infection. 10^6^ of TRBV13-1, TRBV13-2 cells, or a 1:1 mixture of both (mix) were transferred into CB6F1 mice one day prior to infection with RSV. On days 4, 5, and 6 post-infection, lungs were removed, homogenized, and analyzed for IFNγ, TNFα, IL-6 and MIP-1α by multiplex ELISA. Bars represent mean ± SEM from 4 mice/group.

We assessed cytokine levels in the lungs of RSV-infected mice after transfer of TRBV13-1, TRBV13-2 or a 1:1 mix. On day 4, 5 and 6 post-infection, lungs of infected mice were removed and processed for analysis of cytokine levels. On day 4 post-infection, there were significantly higher levels of the pro-inflammatory cytokine interleukin-6 (IL-6) and chemokine macrophage inflammatory protein-1 alpha (MIP-1α) in the lungs of mice that received TRBV13-2 Tg cells alone than in those that received TRBV13-1 Tg cells or the mix (Fig 2, p<0.01). Mice that received TRBV13-2 had increased levels of tumor necrosis factor alpha (TNFα) compared to the mix (p<0.001), but not significantly higher levels than the TRBV13-1 transfer group. There was no significant difference in the level of interferon gamma (IFNγ) between groups. For all cytokines, higher levels were measured in mice who received transfers than in the no transfer control on day 4. The cytokine levels decreased on day 5 and reached levels equal to the no transfer controls by day 6 post-infection. These data show that transfer of only TRBV13-2 Tg cells resulted in higher levels of IL-6 and MIP-1α early after RSV infection compared to transfer of TRBV13-1 Tg cells.

### The TRBV13-2 /TRBV13-1 ratio in the MLN is transfer dose-dependent

**Fig 3:**
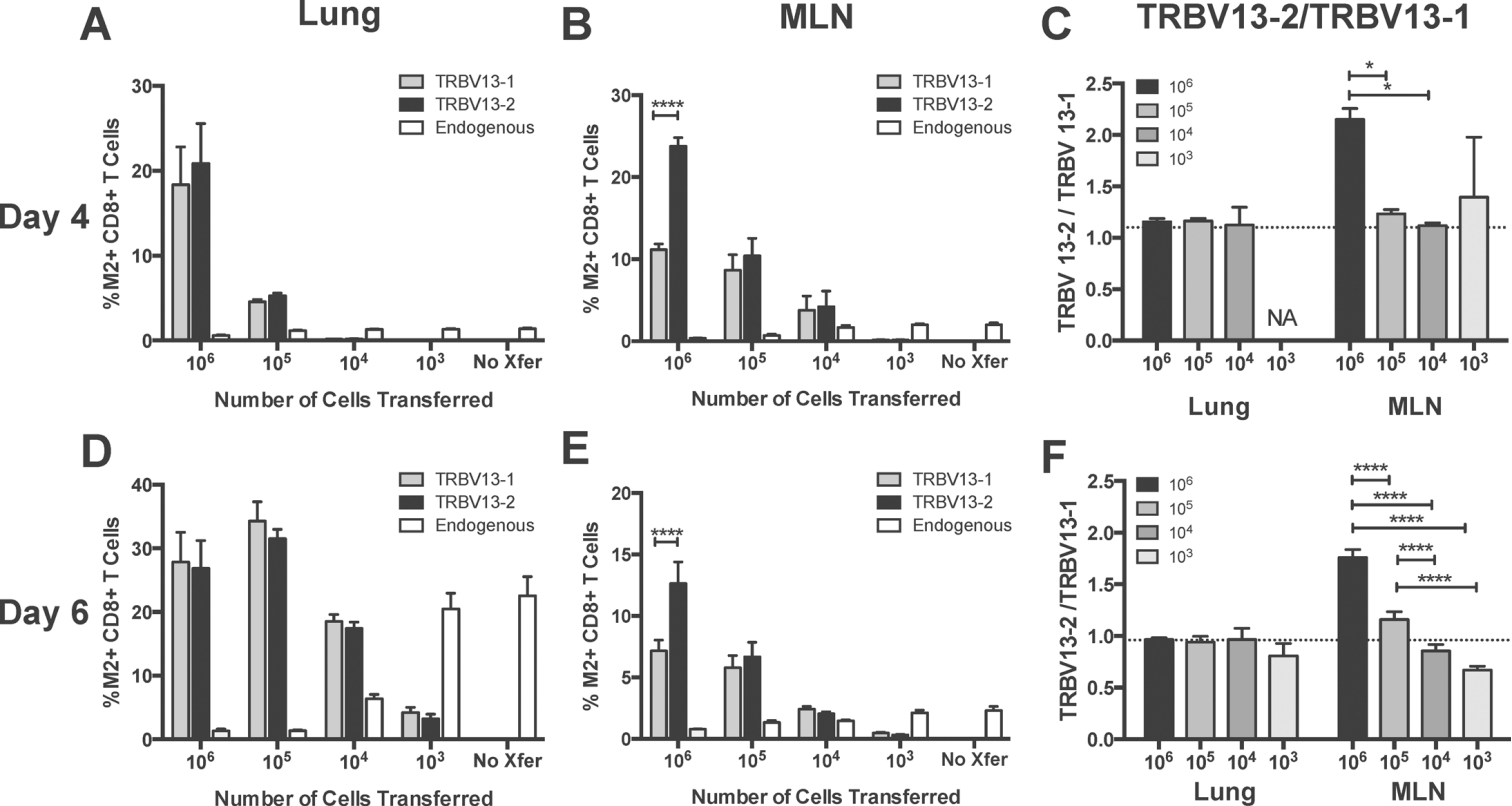
Effects of transfer cell number on TRBV13-2/TRBV13-1 ratio and endogenous CD8+T cell response. The indicted number of a 1:1 mix of TRBV13-1 and TRBV13-2 were transferred into CB6F1 mice one day prior to RSV infection. On **(A-C)** day 4 and **(D-F)** day 6, lungs and mediastinal lymph node (MLN) were collected and the percentages of transferred TRBV13-1, TRBV13-2, and endogenous M2-specific CD8+T cells were determined. **(C, F)** TRBV13-2/TRBV13-1. Dotted line indicates input ratio of TRBV13-2/TRBV13-1. NA indicates that there were not enough events to calculate TRBV13-2/13-1.

We next asked about the dynamics of both M2-specific T cell lines within the same infected host. We evaluated the hierarchy of TRBV13-2 and TRBV13-1 co-transferred at 10-fold dilutions of a 1:1 ratio one day prior to RSV infection. At day 4 and 6 post-infection, lungs and MLN were harvested and the frequency of TRBV13-2 and TRBV13-1 determined using flow cytometry. We were able to distinguish TRBV13-2 and TRBV13-1 from each other and the endogenous response by staining these cells with antibodies against their Vβ chain and the congenic marker Thy1.1. When 10^6^ cells were transferred, there were significantly higher percentages of TRBV13-2 in the MLN (p<0.0001), but similar percentages in the lungs at 4 and 6 days post-infection (Fig 3A and 3B). At all other dilutions, there was no significant difference between the percentages of TRBV13-1 and TRBV13-2 in the MLN and lung. We calculated the TRBV13-2/TRBV13-1 ratio by dividing the percentages of TRBV13-2 by TRBV13-1. In the MLN 6 days post-infection, the TRBV13-2/TRBV13-1 ratio decreases in a dose-dependent manner (Fig 3F) and there is a similar trend at day 4 post-infection (Fig 3C). TRBV13-2 and TRBV13-2 were equally represented in the lung, with a ratio near one for all transfer doses at both time points post-infection. Transfer of high numbers of cells resulted in suppression of the endogenous M2-specific response. However, as the number of cells transferred decreased, the frequency of the endogenous response increased. When only 10^3^ cells were transferred, the endogenous response was similar to the response observed in the no transfer control.

**Fig 4:**
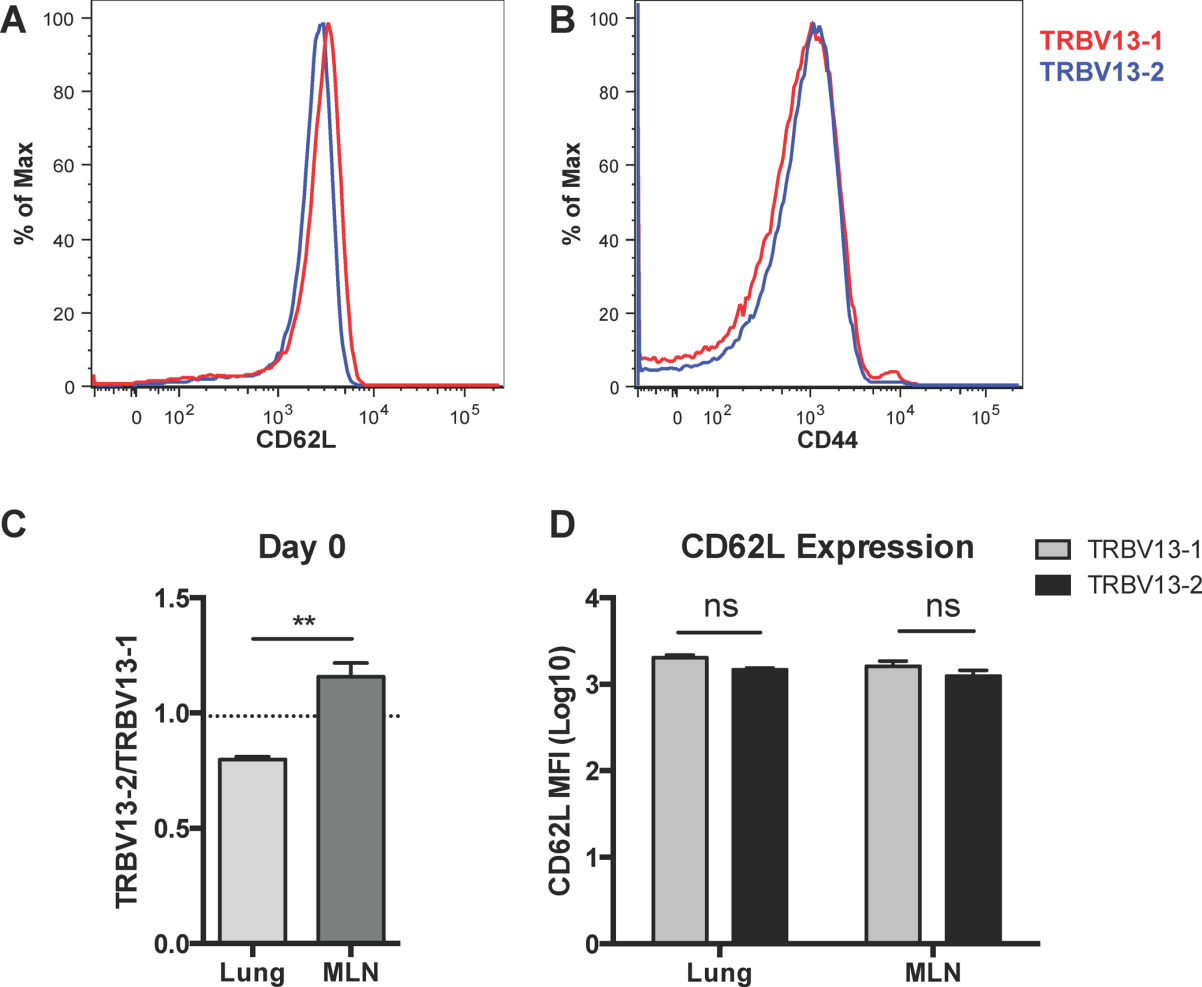
Preferential seeding of TRBV13-2 in the MLN and TRBV13-1 in the lung. 10^6^ of a 1:1 Mix of TRBV13-1 and TRBV13-2 Tg cells were transferred into mice. Seeding of transferred, Thy1.1+ T cells was determined one day later in the lung and MLN. A) Expression of CD62L on TRBV13-2 (blue) and TRBV13-1 (red) before transfer. B) Expression of CD44 on TRBV13-2 and TRBV13-1 prior to transfer. C) Ratio of TRBV13-2/TRBV13-1 in the lung and MLN one day after transfer. Dotted line indicates input ratio of TRBV13-2/TRBV13-1. D) CD62L expression on TRBV13-1 and TRBV13-2 Tg cells in the lung or MLN one day after transfer. Bars represent mean±SEM of five samples of 2-pooled mice each. **p<0.01 by paired t test.

To determine if TRBV13-2 selectively homed to the MLN following transfer, we analyzed the composition of T cells in the lung and MLN one day following transfer of 10^6^ of a 1:1 mixture of Tg cells. We found that transferred, Thy1.1+ TRBV13-2 preferentially seeded the MLN while TRBV13-1 preferentially seeded the lung (Fig 4C, p<0.01). CD62L is important for entry into the secondary lymphoid organs, so we hypothesized that CD62L expression would be higher on TRBV13-2 cells leading to increased seeding in the MLN. Interestingly, we found that CD62L expression was slightly higher on TRBV13-1 Tg cells than TRBV 13–2 Tg cells prior to transfer (Fig 4A). This trend was also evident on day 1 post-transfer in both the lung and MLN, but the difference was not significant (Fig 4D). There were also similar levels of CD44 and CD127 expression on TRBV13-1 and TRBV13-2 cells (Fig 4B and not shown). Despite this slightly higher level of CD62L on TRBV13-1 cells, TRBV13-2 preferentially seeded the MLN. Due to low event counts, the seeding of the MLN and lung was not able to be assessed for groups receiving a lower transfer dose.

**Fig 5:**
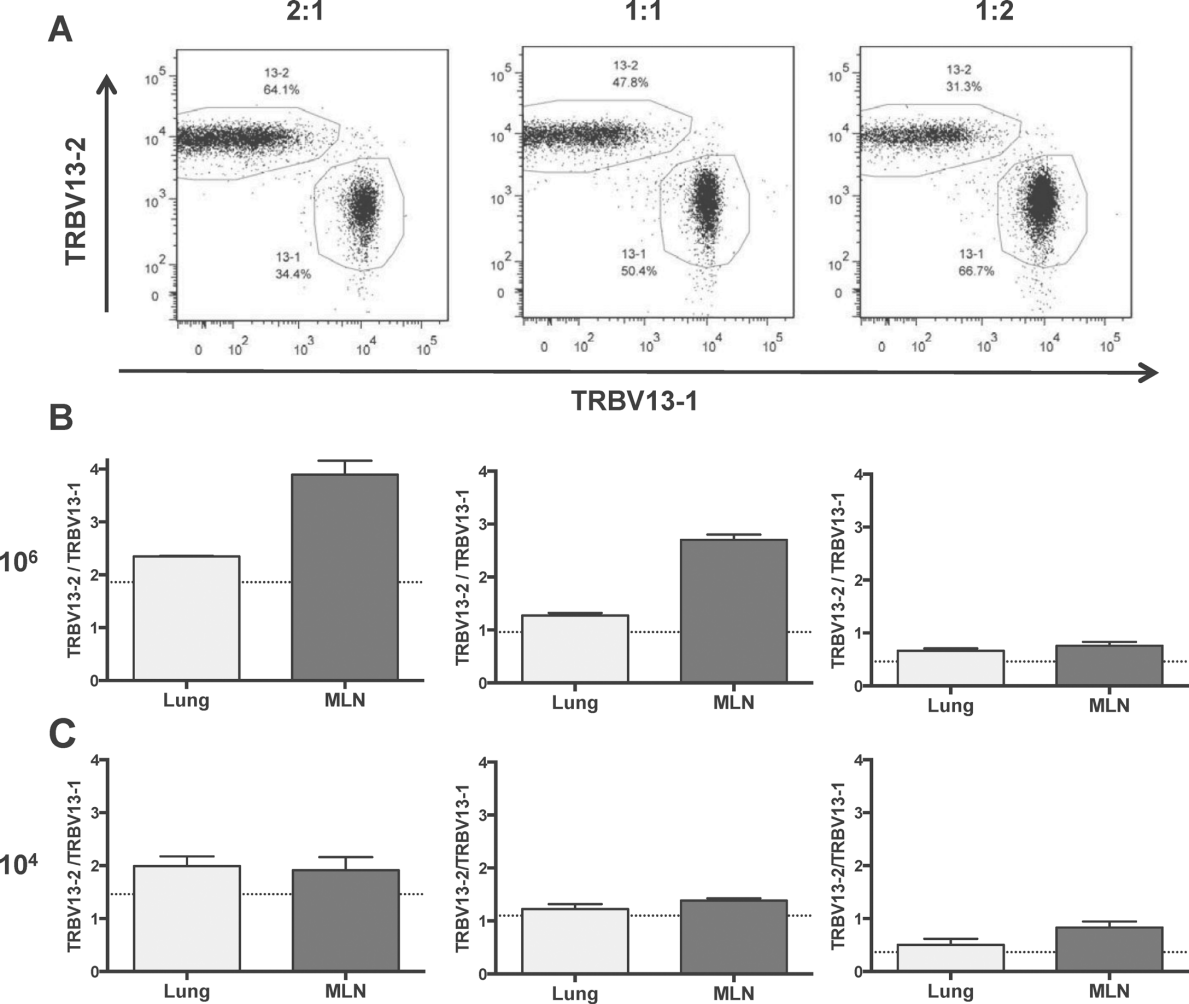
Different input TRBV13-2/TRBV13-1 ratios affect the magnitude of TRBV13-2 skewing. 10^6^ or 10^4^ cells were transferred into mice at input ratios of 2:1, 1:1, and 1:2 of TRBV13-2:TRBV13-1 and mice were infected with RSV one day later. (A) Flow cytometry plots showing the input frequency for TRBV13-2 and TRBV13-1 at different ratios for the 10^6^ cell number transfer. (B and C) TRBV13-2/TRBV13-1 ratio on day 6 post-RSV infection in the lungs and MLN of mice who received different TRBV13-2/TRBV13-1 ratios. Dotted line indicates input ratio of TRBV13-2/TRBV13-1.

To further examine the relationship between TRBV13-2 and TRBV13-1 when co-transferred, we transferred 10^6^ or 10^4^ total transgenic cells in three different TRBV13-2/TRBV13-1 ratios: 2:1, 1:1 and 1:2 (Fig 5). In this experiment, the same trend was seen with a higher TRBV13-2/TRBV13-1 ratio when higher cell numbers were transferred. As seen previously, the TRBV13-2 skewing was observed in the 1:1 mix. This skewing was exacerbated when a 2:1 ratio was transferred and mitigated at lower transfer numbers. Regardless of transfer ratio, there was more TRBV13-2 skewing in the MLN than in lung consistent with experiments utilizing a 1:1 mixture of both lines.

### Proliferation is transfer dose-dependent, but does not differ between Tg lines

**Fig 6:**
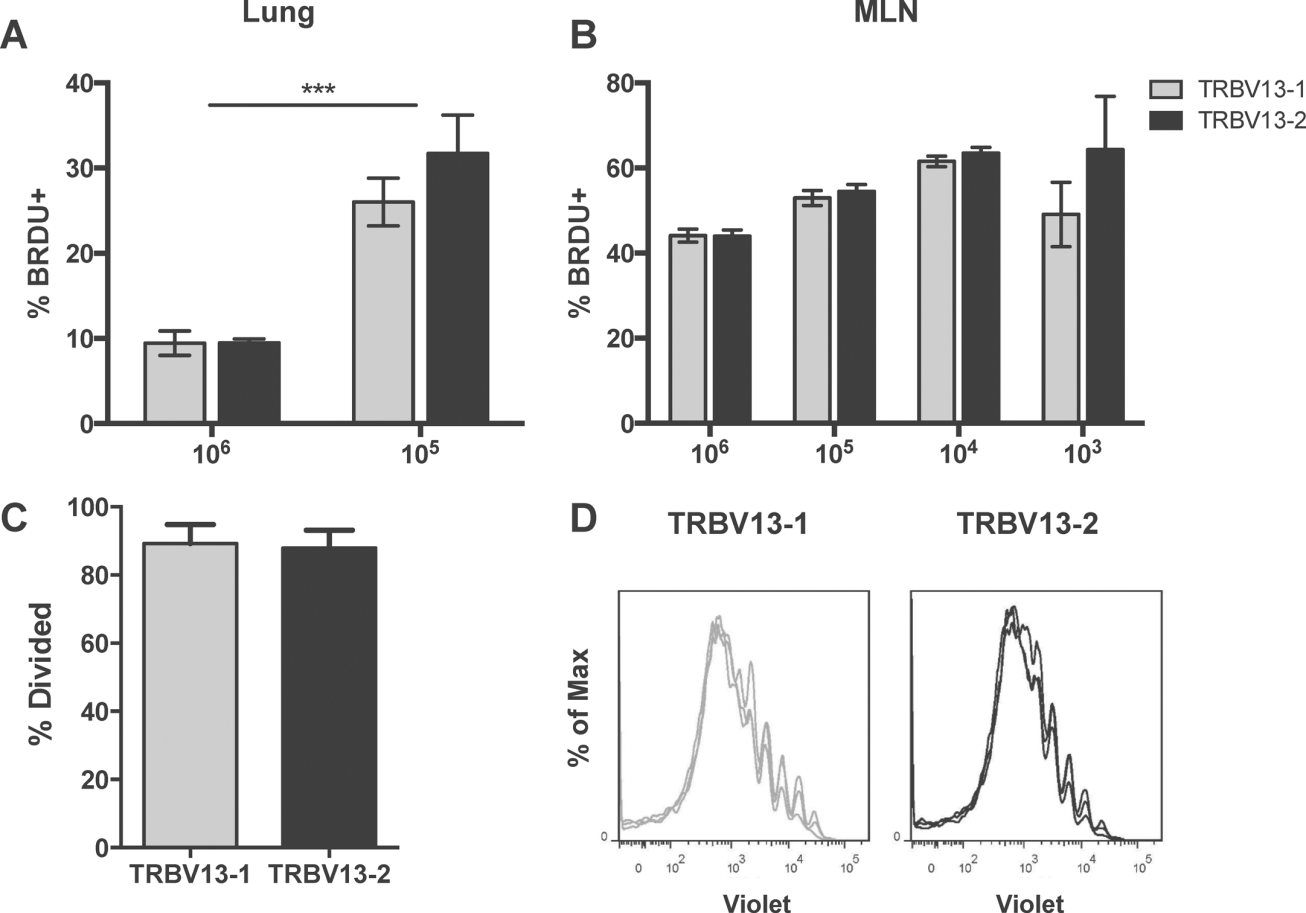
Proliferation is dependent on transfer number, but does not differ between TRBV 13–1 and TRBV13-2. (A and B) 1:10 dilutions of a 1:1 ratio of TRBV13-1:TRBV13-2 cells were transferred 1 day prior to RSV infection. Four days post-infection, the percentage of proliferating cells in the lung (A) and MLN (B) were identified by *in vivo* incorporation of BrdU during a two-hour incubation. **(**C and D) violet labeled TRBV13-1 and TRBV13-2 were co-transferred into CB6F1 mice prior to infection with RSV. Proliferation profiles of TRBV13-1and TRBV13-2 (C) in the MLN were evaluated at day 4 post-infection and percentage of dividing cells was determined (D).

Skewing towards TRBV13-2 in the MLN with larger transfer numbers could be explained by faster proliferation by the TRBV13-2 transgenic cells. To address this possibility, we evaluated *in vivo* and *in vitro* proliferation of the two Tg lines. To evaluate *in vivo* proliferation, we transferred 10-fold dilutions of a 1:1 mix of TRBV13-2 and TRBV13-1 one day prior to RSV infection. On day 4 post-infection, we administered 2mg BRDU IP to identify actively proliferating cells. Two hours post-BRDU administration, lungs and MLN were harvested and the percentages of cells with BRDU incorporation were analyzed. Both lines were found to proliferate equally *in vivo* (Fig 6A and 6B). Transfer of low numbers of cells resulted in significantly higher percentages of BRDU-incorporating cells in the lungs. (Fig 6A). In the MLN, there was a trend towards increased proliferation with decreasing cell numbers, but it was not significant (Fig 6B). To confirm this finding, we evaluated proliferation in TRBV13-2 and TRBV13-1 co-transfers by violet dye dilution. Tg CD8+ T cells were labeled with CellTrace Violet viability dye and 10^6^ of a 1:1 mix of TRBV13-2 and TRBV 13–1 cells were transferred into CB6F1 mice one day prior to RSV infection. Four days post-infection, MLN were harvested and analyzed for dilution of violet dye. TRBV13-2 and TRBV13-1 cells showed similar proliferation profiles and percentages (Fig 6C and 6D). Analysis of the dividing cells showed that both cell lines exhibited similar high rates of proliferation of almost 90% (Fig 6C). Since there were no differences in proliferation between lines, we assessed apoptosis in co-transfers of 10^6^ TRBV13-2 and TRBV13-1 by AnnexinV binding analysis. We found no difference in the apoptosis rates of TRBV13-2 and TRBV13-1 Tg cells (data not shown). Collectively, these data show that despite higher percentages of TRBV13-2 in the MLN of mice who received 10^6^ Tg cells, we found no measurable difference in proliferation or apoptosis between the Tg T cell lines.

### Cell transfer number influences the Transgenic T cell phenotype

**Fig 7:**
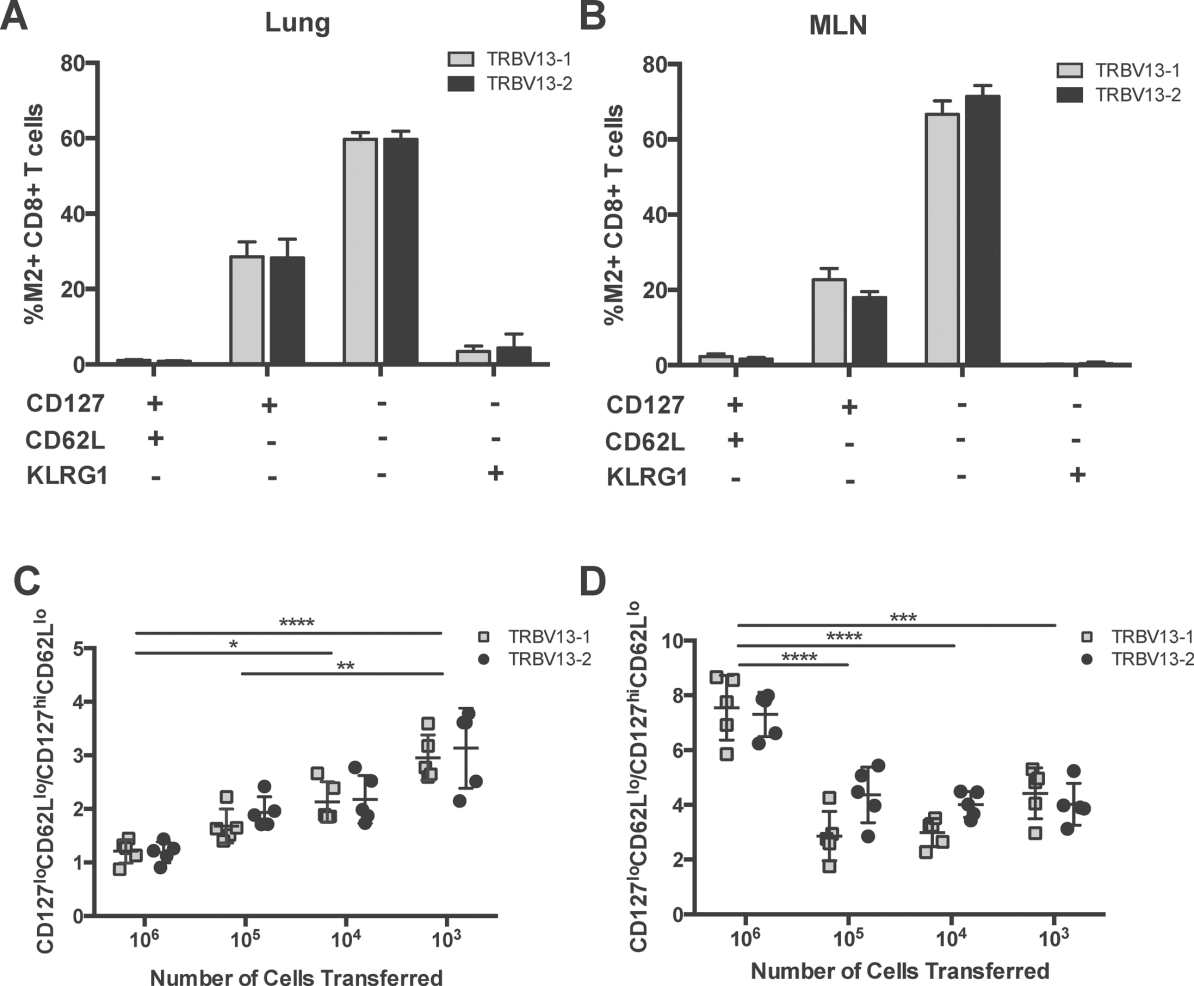
Cell transfer number affects the Tg T cell phenotype. Different numbers of a 1:1 mixture of TRBV13-2:TRBV13-1 cells were transferred 1 day prior to RSV infection. On day 6 post-infection, we assessed the phenotype of the transferred Tg T cells based on expression of CD62L, CD127, and KLRG1. The percentage of Tg CD8+T cells of each phenotype in the 10^4^ transfer group are in the lungs (A) and MLN (B) are shown. The CD127^lo^, CD62L^lo^; KLRG1^lo^ to CD127^hi^, CD62L^lo^; KLRG-1 ^lo^ ratio in the lungs (C) and MLN (D) was determined. Bars represent mean±SEM.

Next, we sought to determine if there were phenotypic differences between TRBV13-2 and TRBV13-1. Different numbers of a 1:1 mixture of TRBV13-2 and TRBV13-1 were transferred into CB6F1 mice one day prior to RSV infection. Six days post-infection, lungs and MLN were harvested and CD8+ T cells were analyzed for expression of CD127, CD62L, and KLRG1 and categorized into 4 categories CD127^hi,^ CD62L^hi^, KLRG1 ^lo^; CD127^hi^, CD62L^lo^; KLRG-1 ^lo^. CD127^lo^, CD62L^lo^; KLRG1^lo^ and CD127^lo^, CD62L^lo^ and KLRG1^hi^ cells were identified as KLRG1+ effectors. TRBV13-2 and TRBV13-1 Tg cells had similar phenotypes at all cell transfer numbers (Fig 7A and 7B). To compare the phenotype across different input cell numbers, we calculated the CD127^lo^, CD62L^lo^; KLRG1^lo^ to CD127^hi^, CD62L^lo^; KLRG-1 ^lo^ ratio. As the number of cells transferred decreased, the CD127^lo^, CD62L^lo^; KLRG1^lo^ to CD127^hi^, CD62L^lo^; KLRG-1 ^lo^ ratio increased in a dose-dependent manner in the lungs (Fig 7C). In the MLN, this ratio was significantly higher at 10^6^ than at all other cell transfer numbers (p<0.001, Fig 7D). These results indicate that CD8+ T cell phenotype following infection depends on the number of cells transferred, with both lines responding identically. In addition, to day 6 post-infection, we attempted to evaluate the memory phenotype at day 30 post-infection. However, we found that cells adoptively transferred from these Tg cell lines do not establish a memory population following RSV infection, possibly due to an inability to upregulate of CD127 after effector expansion (S1 Fig). Compared to the endogenous M2-specific CD8+ T cells, both TRBV13-1 and TRBV13-2 in the MLN have lower levels of CD127 expression on day 6 post-infection (S1 Fig). A similar phenomenon has previously been described in the adoptive transfer experiments of SMARTA CD4 Tg lines and CD8 Tg lines specific for the P1A tumor antigen which fail to generate memory cells under certain conditions despite displaying early effector function[14, 15].

## Discussion

In this study, we have characterized the *in vivo* characteristics of two TCR Tg lines targeting the RSV K^d^M2_82-90_ epitope on a CB6F1 background. When TRBV13-2 and TRBV13-1 are adoptively transferred, they exhibit similar abilities to control viral load, but the transfer of TRBV13-1 Tg T cells causes more morbidity. Interestingly, when high numbers of TRBV13-2 and TRBV13-1 cells are co-transferred into recipient mice prior to RSV infection, they establish a hierarchy in the MLN with TRBV13-2 cells being numerically dominant over TRBV13-1. At lower cell numbers, the number of each subset are equalize and begin to skew towards TRBV13-1. Despite differences in the frequency of these lines, we found no difference in proliferation or phenotype between TRBV13-2 and TRBV13-1 at all cell transfer numbers. However, transfer dose does affect the phenotype and proliferation of both of these Tg T cell lines.

Despite measuring no difference in proliferation or apoptosis, the TRBV13-2 Tg T cells are numerically dominant over the TRBV13-1 T cells in the MLN when 10^6^ cells are transferred. This dominance of TRBV13-2 may be due to preferential seeding of TRBV13-2 in the MLN, while TRBV13-1 preferentially seeded the lung. However, this dominance is dose-dependent and the TRBV13-2 and TRBV13-1 remain at similar numbers when lower cell numbers are transferred. CD8+ T cells have been shown to compete with other CD8+ T cells with the same epitope specificity as well as CD8+ T cells specific for different MHC:peptide complexes [16–18]. Since the decrease in the TRBV13-2 dominance coincides with the increase in the endogenous response, a possible explanation for the change in hierarchy is that the TRBV13-2 Tg T cells are more susceptible to competition from the endogenous response. This competition may be from other endogenous M2-specific CD8+ T cells or T cells specific for other epitopes. This skewing towards TRBV13-2 was only seen in the MLN and not in the lung. However, we found that TRBV13-1 preferentially seeded the lung, and thus were numerically dominant in the lung at the time of RSV infection. Thus, even finding an equal ratio in the lung several days post-infection, may indicate skewing towards TRBV13-2 in the lung. These data demonstrate that even TCR Tg lines specific for the same epitope can respond differently to the *in vivo* environment after infection.

In our previous study, we found a slight but significant difference in proliferation between TRBV13-2 and TRBV13-1 when 5x10^6^ TRBV13-2 or TRBV13-1 cells were adoptively transferred into BALB/c mice. However, in this study we found no significant difference in proliferation between TRBV13-2 and TRBV13-1 when transferred into CB6F1 mice. Here, we have substantially lowered the number of transferred cells, and have improved upon our ability to identify adoptively transferred cells by the inclusion of a congenic marker where the previous study identified transferred cells based on tetramer binding. While the transfer of large numbers of transgenic cells nearly eliminates the endogenous response, we cannot rule out that the endogenous response, or the high numbers of transferred cells, contributed to the slight difference we observed in the previous studies. We have additionally evaluated BRDU incorporation during co-transfer experiments in these studies to eliminate mouse-to-mouse variability as a potential source of error. These differences likely account for the discrepancies between our previous and current findings. Additionally, we cannot rule out that slight proliferation differences do exist between these lines, that we are unable to detect by these methodologies.

In this study, we found that phenotype and frequency of cells undergoing proliferation depends on precursor frequency. The ratio of CD127^lo^CD62L^lo^KLRG1^lo^ to CD127^hi^CD62L^lo^KLRG1^lo^ ratio rose when lower numbers of cells were transferred. Expression of phenotypic markers CD62L, CD127 and functional ability have been previously shown to depend on the input frequency of OT-I TCR CD8T transgenic T cells [19–21]. Additionally, the transfer of lower number of cells resulted in a greater percentage of proliferating cells. This consistent with previous finding, which show that transgenic precursor frequency affects the percentage of proliferating cells [22]. Our data expand upon these findings and demonstrate that these findings are applicable to other TCR Tg T cell systems and in scenarios when multiple T cell lines are co-transferred.

In conclusion, we have shown that two TCR Tg lines specific for the RSV K^d^M2_82-90_ epitope have similar *in vivo* properties including ability to control viral loads, phenotype, and proliferation in the CB6F1. However, we found that high transfer numbers skewed the hierarchy of these cells in the MLN. Different cell transfer numbers also affected phenotype and proliferation capacity, emphasizing the importance of considering transfer dose in adoptive transfer experiments, particularly when comparing to endogenous responses with a considerably lower precursor frequency.

## Supporting Information

S1 Fig Transferred M2-specific Tg lines have lower expression of CD127 than endogenous M2-specific CD8 T cells. 10^6^ and 10^5^ of a 1:1 mixture of TRBV13-2:TRBV13-1 cells were transferred 1 day prior to RSV infection. On day 6 post-infection, we assessed the expression of CD127 on TRBV13-1 Tg cells (red), TRBV13-2 Tg cells (blue) and endogenous M-specific CD8 T cells (black).
(TIFF)
