# Supplementary figures and images for: Phenotype and Hierarchy of Two Transgenic T Cell Lines Targeting the Respiratory Syncytial Virus KdM282-90 Epitope Is Transfer Dose-Dependent
Source: PLoS One. 2016 Jan 11;11(1):e0146781. doi: 10.1371/journal.pone.0146781 (PMC4708989; doi:10.1371/journal.pone.0146781)

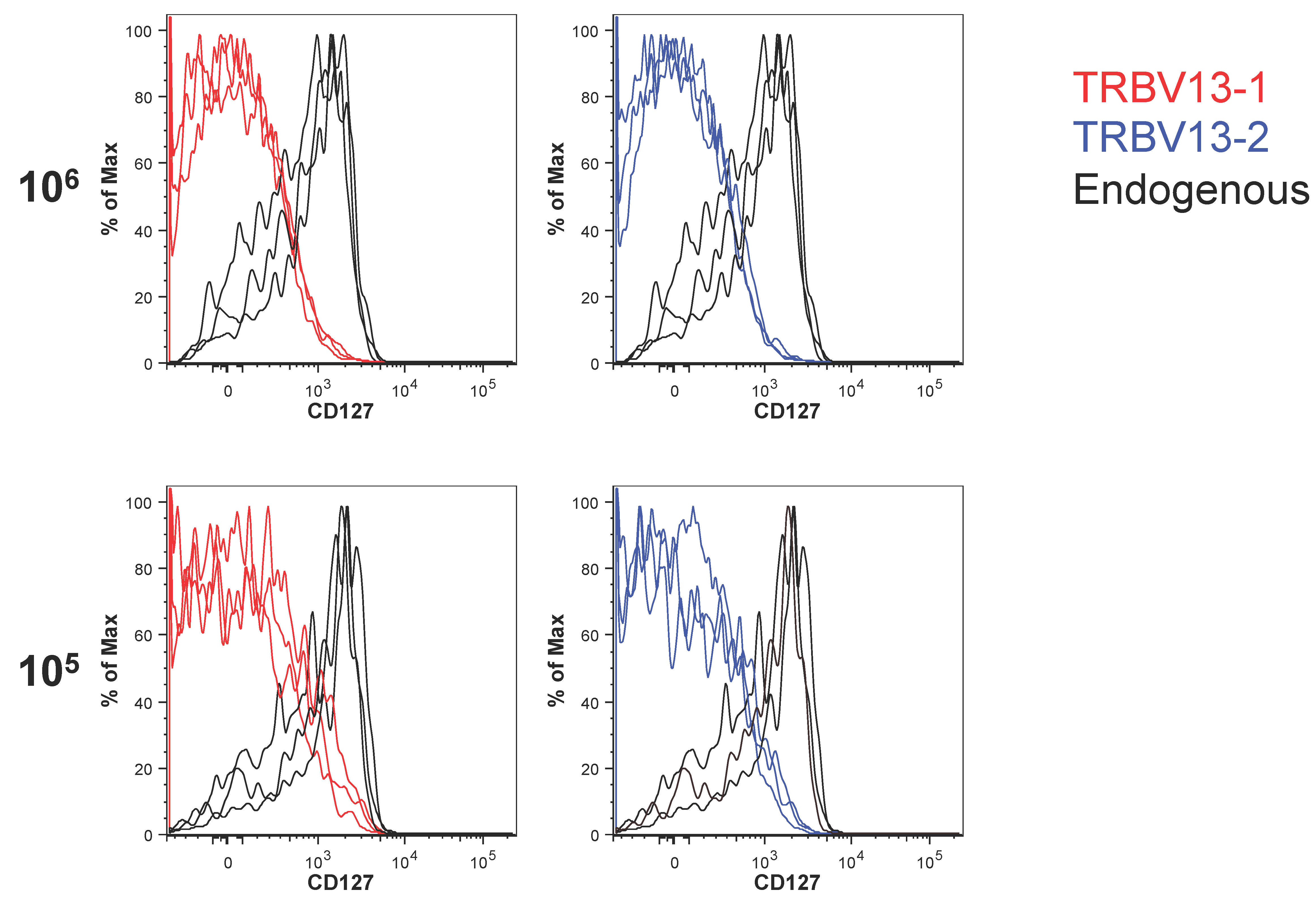

Supplement: S1 Fig — 106 and 105 of a 1:1 mixture of TRBV13-2:TRBV13-1 cells were transferred 1 day prior to RSV infection. On day 6 post-infection, we assessed the expression of CD127 on TRBV13-1 Tg cells (red), TRBV13-2 Tg cells (blue) and endogenous M-specific CD8 T cells (black). (TIFF) [file pone.0146781.s001.tiff]
